# Editorial: Quality Assessment Across Disciplines in Head and Neck Cancer Treatment

**DOI:** 10.3389/fonc.2021.636622

**Published:** 2021-02-19

**Authors:** Jan B. Vermorken, Dirk Van Gestel

**Affiliations:** ^1^ Department of Medical Oncology, Antwerp University Hospital, Edegem, Belgium; ^2^ Faculty of Medicine and Health Sciences, University of Antwerp, Antwerp, Belgium; ^3^ Department of Radiation Oncology, Institute Jules Bordet, Université Libre de Bruxelles, Brussels, Belgium

**Keywords:** head and neck cancer, squamous cell, multidisciplinary team approach, quality assessment, quality assurance

## Introduction

Quality assessment is indispensable in all areas of cancer care, and that is certainly true for head and neck cancer. The increased complexity of diagnosis and the multimodal and multiprofessional treatment and expanding (financial) burden to the health care system leads to concern whether new knowledge is effectively transferred to all patients. Moreover, there are major geographical differences in accessibility of radiotherapy equipment and anti-cancer drugs around the globe. Impaired quality of care results in compromised quality of life or sometimes even in impaired survival. Quality assurance must cover the entire patient care process from the diagnosis to the therapeutic decision and for that the multidisciplinary team (MDT) meetings are playing a crucial role.

## Topics of Interest

In the present Research Topic on “Quality Assessment Across Disciplines in Head and Neck Cancer Treatment” leaders in the head and neck oncology field discuss a variety of topics:

1. Quality of Care at a European Level. Based on a multidisciplinary and expert-based consensus process the main quality indicators for quality of care are considered to be the availability of a formalized multidisciplinary team, participation in clinical and translational research, timeliness of care, high quality of surgery and radiotherapy and adequate pathology reporting. These quality indicators were retrospectively tested in four European countries in patients treated in the years 2009–2011 (see Trama et al.). For head and neck cancer, the quality of care did not reach the optimal standard in most of these countries. Patients frequently were found diagnosed at an advanced stage, a high proportion showed delays in starting therapy (especially for radiotherapy) and only a limited use was made of multimodal therapy. One of the suggested options to improve these disappointing observations is referring patients to specialized centers or networks including specialized centers.
2. Quality Indicators in Belgium. In line with these observations is a nation-wide study in Belgium of 9,245 patients with squamous cell head and neck cancer diagnosed between 2009 and 2014. Leroy et al. reported that four quality indicators of diagnosis and staging (dedicated imaging studies, use of PET-CT, TNM reporting and interval between diagnosis and start of therapy) varied widely among hospitals in Belgium, clearly shown in room for improvement.
3. The Importance of MDT Meetings. The composition and respective roles of the different disciplines in the MDT meetings are described by Taberna et al., highlighting the role of the head and neck cancer specialized nurse, the dietitian, the speech and language pathology expert and the onco-geriatrician. They also indicate the importance of dental care and psychosocial support, and stress the importance of integrating clinical and translational research teams in the MDT meetings.
4. Diagnostic Pathology. Accurate diagnosis is key to provide a high quality clinical service for patients with head and neck cancer. Treatment should not be provided unless a histological diagnosis of cancer is made. For this to be accurate, both the laboratory and reporting service must be quality assured. Sloan and Robinson stress the importance of clinical audits as a method to improve the healthcare services, while digital pathology has the potential to bring about improvements in the safety, quality and efficiency of a cellular pathology department. Artificial intelligence systems most likely will infiltrate this arena, also in head and neck pathology. Many innovations are expected to be implemented and within that context quality assurance for molecular testing, including for biomarkers, seems indispensable.
5. Biomarkers. Economopoupou et al. provide a descriptive and detailed review of validated biomarkers in squamous cell head and neck cancer. HPV DNA/p16, PET imaging and PDL-1 are validated diagnostic and prognostic/predictive biomarkers currently used in clinical practice. The utility of several emerging biomarkers, such as e.g.; skeletal muscle mass and next generation sequencing, and their usefulness in the future are thought to depend on accuracy, reproducibility and reliability.
6. PET-CT Imaging. A more in depth quality assessment in FDG-PET-CT imaging is given by Van den Wyngaert et al., summarizing the recent technical breakthroughs in PET-CT scan design and describes the existing quality assessment frameworks with a focus on applications in head and neck cancer.
7. Best Practice in Surgical Treatment. It is already known for quite some time that high-volume hospitals provide a lower long-term mortality and this also holds for the number of patients that a physician sees (the more the better). Also center criteria like participation in clinical trials and transparency of clinical outcome are considered mandatory for high quality patient care. Simon et al. in their manuscript highlight that sticking to guidelines, avoiding delays in treatment (primary as well as adjuvant), pretreatment multidisciplinary evaluation, elective neck dissection yield of ≥18 lymph nodes and obtaining a negative surgical margin all are associated with improved survival. They describe the best practice guidelines for the surgical treatment of cancers of the sino-nasal tract, skull base, aerodigestive tract in the neck.
8. Radiotherapy Quality Assurance. Quality assurance has been taken seriously first by the radiation oncologists and is most advanced among the different disciplines involved in the treatment of patient with head and neck cancer. Because the actual radiotherapy procedures in head and neck cancer have become so complex and so precise rigorous quality assurance for all the different steps in the radiation process is essential to deliver the right dose to the right place in order to reach the best oncologic outcome with the least toxicity for the organs at risk. Again, as described for the best practice in surgery, outcome results in large volume centers are better than in low volume centers. Van Gestel et al. consider the reason for this finding likely multifactorial, including not only the quality of the radiotherapy planning and delivery, but also the quality and accuracy of other steps involved in tumor staging (e.g.; pathology, imaging) and treatment.
9. Best Practice in Systemic Therapy. Oosting and Haddad also highlight the importance of the MDT meetings within the context of quality assurance. Treatment in a multidisciplinary team is essential and improves outcome. Quality assurance in daily practice should aim at guideline implementation, specialization and multidisciplinary care and should pay attention especially to the older patients, patients with comorbidity and patients from lower socio-economic classes. With respect to the selected treatment, the preparation of the drug, the delivery to the patient (administration and dosage, giving the correct dose intensity), and handling the side effect in a timely manner are crucial in obtaining the best outcome. Several trials, both prospectively and retrospectively have indicated that suboptimal dosing of chemotherapy is leading to impaired outcome. Monitoring of outcome and benchmarking can be strong incentives to assess and improve quality of care.
10. Statistical Issues. Fortpied and Vinches elegantly explain what the tasks are of the medical statistician when performing a clinical study. They stress the importance of a good communication between the statistician and the research physician. The complexity of head and neck cancer as being a heterogeneous disease, the integration of new therapies in both the primary disease setting and the recurrent/metastatic disease setting, the change in head and neck cancer population and the comorbidities add to the already existing challenges when performing clinical trials.
11. Follow-Up of Head and Neck Cancer Survivors. How intensive should this be. The impact on overall survival has been equivocal. However, no randomized trial exploring this question has been done. Based on literature data, Szturz et al. formulated arguments in favor of and against intensive follow-up and propose a compromise in which in specific clinical situations periodic imaging can be justified.
12. Supportive Care. Bonomo et al. indicate that the quality of supportive measures are essential as “supportive care makes excellent cancer care possible”. The quality of supportive care makes it possible to administer drug treatment according to planned dose intensity. Moreover, when the primary treatment concerns surgery, prevention of infections and pain control after surgery as well as a rapid rehabilitation (early oral feeding after surgery) are important items. With respect to radiotherapy with or without systemic therapy, prevention of oral and oropharyngeal mucositis and radiodermatitis, giving nutritional support, apply swallowing exercises, giving adequate pain therapy and helping patients with psychosocial support are all important items. An early involvement of a supportive and palliative team is a central issue to allow for a better patient information and care. For patients with recurrent/metastatic disease such teams will also help to avoid administering chemotherapy in the last period of life.

## Author Contributions

All authors listed have made a substantial, direct, and intellectual contribution to the work and approved it for publication.

## Conflict of Interest

The authors declare that the research was conducted in the absence of any commercial or financial relationships that could be construed as a potential conflict of interest. 

